# The Effect of Various Medicines upon the Milk Secretion

**Published:** 1882-07

**Authors:** 


The Effect of Various Medicines upon the Milk Secretion.—This subject, which should be of much interest to veterinarians, has been recently studied by Dr. Max Stumpf, of Munich (*Archivs für Klinische Medicine*).

He comes to the following conclusions:

I. As regards changes in the quantity of milk.

1. Iodide of potassium lessens the amount.
2. Alcohol, morphine and lead do not affect it.
3. Saticylic acid lessens it slightly.
4. Pilocarpin does not promote milk secretion.

II. Changes in quality.

1. Iodide of potassium causes a diminution in all the ingredients of the milk.
2. Alcohol increases the relative amount of fat.
3. Salicylic acid increases the amount of sugar.
4. Lead, morphine and pilocarpin do not affect the quality.

III. Regarding the passage of medicine into the milk.

1. The iodine of iodide of potassium goes rapidly into the milk, and disappears very soon when medication is stopped. In vegetable feeders the iodine is eliminated in the milk slowly.
2. Alcohol in herbivora does not pass into the milk.
3. Lead goes into the milk in very slight traces.
4. Salicylic acid passes into milk in slight amount.

